# A conversational AI toolkit for citizen deliberation in party primary elections

**DOI:** 10.12688/openreseurope.24102.1

**Published:** 2026-06-13

**Authors:** F. Ramón Villaplana, Michal Malý

**Affiliations:** 1 Political Science, Social Anthropology and Public Finance, Universidad de Murcia, Murcia, Region of Murcia, 30008, Spain; 2 Department of Political Science, Charles University Institute of Political Studies, Prague, Prague, 158 00, Czech Republic

**Keywords:** voting advice; primary elections; conversational AI; democratic deliberation; digital tools; citizen science

## Abstract

Primary elections within political parties are increasingly consequential for democratic outcomes, yet voters –both party members and the general public– frequently lack systematic, comparable and trustworthy information about the candidates competing for nomination. Traditional voter information tools, such as Voting Advice Applications, focus on programmatic alignment in general elections and rarely address the specific demands of intraparty contests, where electability, leadership style, integrity, and organisational capital matter alongside policy positions. The recent availability of large language models with web-search capabilities offers an opportunity to develop accessible, adaptable and free analytical tools that systematise public information about candidates while preserving voter autonomy.

We developed PRIMARYCAN, an open-source prompt-based toolkit consisting of three components: (1) a main analytical prompt that generates structured candidate profiles in a default accessible mode and an optional professional mode; (2) an external evaluator prompt for auditing outputs in a different model; and (3) a methodological guide. The toolkit produces structured analyses that are comparable across candidates, traceable to public sources, and adaptable to user expertise. Six analytical blocks cover identity and trajectory, discourse and communication, capabilities and performance, consistency and integrity, political capital and electoral potential and (optionally) advanced behavioural indicators.

PRIMARYCAN includes safeguards against common pitfalls in algorithmic political analysis: voting verdicts are prohibited; criteria symmetry is enforced; behavioural patterns replace clinical psychometric labels; hindsight bias safeguards are mandatory in retrospective analyses. The toolkit illustrates that conversational AI, when carefully scaffolded with explicit ethical and epistemic constraints, can serve as a useful intermediary for democratic deliberation in primary elections. We discuss limitations (model variability, hallucination risk, structural limits of self-evaluation), pending empirical validation with real users, and pathways for adaptation to other electoral contexts.

## 1. Introduction

Primary elections –the internal party processes by which political organisations select their leaders or candidates for general elections– have grown in importance across European and global democracies over the past two decades ( Cross & Blais, 2012; Sandri, Seddone, & Venturino, 2015; Cross et al., 2016). The trend is uneven but persistent: more parties open candidate selection to broader electorates, more decisions of consequence are made within parties before they reach general voters, and the legitimacy of party leadership increasingly depends on the perceived fairness and informativeness of these internal contests ( Scarrow et al., 2022).

Yet the information environment in which the primary *selectorate* operates remains structurally weaker than in general elections. General-election campaigns are covered intensively by professional media, scrutinised by opposition parties with adversarial incentives and supported by an ecosystem of civic tools –Voting Advice Applications, candidate comparison platforms, fact-checking agencies, etc.– that aggregate and translate political information for the general public ( Snyder & Strömberg, 2010; Gemenis & Rosema, 2014; Amazeen, 2020; Ferrer, 2023). Primary elections, in contrast, often unfold in compressed timeframes, with media coverage focused on horse-race dynamics rather than substantive candidate evaluation, with opposition coming from fellow partisans rather than external adversaries and with few or no dedicated tools to help voters assess candidates beyond their public appearances.

This asymmetry has consequences. Party members –a small fraction of the general electorate, but a politically committed and highly mobilised one– make decisions of cascading importance: the leader or candidate selected in a primary often becomes the party’s face for years, defines its strategic direction, and may eventually become a head of government. General voters, in turn, find themselves choosing between candidates whose selection processes they did not monitor and whose internal trajectories they cannot easily reconstruct retrospectively. The result is an informational deficit at the point in the political cycle where small electorates make decisions of larger impact.

This paper describes PRIMARYCAN, a software tool designed to address this deficit. The tool is a conversational AI prompt –an open-source set of instructions that, when pasted into a large language model with web-search capabilities (such as Claude, ChatGPT or Gemini), produces structured candidate analyses tailored to a specific primary election. The tool is freely usable, adaptable to any political context, multilingual and designed around principles of voter autonomy, evidence-based rigor and transparency. It does not tell voters whom to vote for. It systematises publicly available information about each candidate, organises it around dimensions that matter for the decision, and returns analytical agency to the user.

The toolkit consists of three components: a main analytical prompt, an external evaluator prompt for auditing outputs and a methodological guide. It has been iteratively designed through six successive versions, with controlled testing across five distinct cases.

The rest of this paper is structured as follows. Next section situates the tool in relation to existing literature on voter information, voting advice applications, intraparty democracy and AI-assisted deliberation. Third section describes the implementation of the toolkit, including its architecture, design principles, and ethical safeguards. Section four discusses limitations, risks and pending empirical validation. The last section concludes with implications and pathways for further development and the integration of this tool into more complex systems such as AI agents.

## 2. Background and motivation

### 2.1 The information gap in primary elections

Voter information in elections has long been studied as a determinant of decision quality, electoral participation and democratic legitimacy ( Lupia, 1994; Hansen, & Pedersen, 2014; Dahlgren, 2018). The classical finding is that voters compensate for information deficits with cognitive shortcuts –partisan identification, candidate appearance, endorsement cues– which can produce reasonable decisions in stable contexts but fail in unfamiliar ones ( Lazarsfeld, Berelson & Gaudet, 1948; Popkin, 1991). Primary elections are precisely such unfamiliar contexts for many voters: party affiliation does not differentiate the candidates, ideological cleavages are often subtle or absent and unfamiliar names compete on grounds –leadership, electability, integrity– that are harder to evaluate than policy positions.

The literature on intraparty democracy has documented this challenge from multiple angles. Hazan and Rahat (2010) emphasise that the choice of selection method shapes who can win primaries and on what grounds, but the information available to selectors typically lags behind these structural changes. Cross and Pilet (2015) note that the spread of more inclusive selection methods has not been accompanied by a proportional development of informational infrastructure for participants. Sandri, Seddone, and Venturino (2015) document how primary campaigns differ from general election campaigns in ways that can disadvantage less-known candidates and benefit those with established media presence, regardless of substantive merits.

### 2.2 Voting advice applications and their limits

Voting Advice Applications (VAAs) are the most widely deployed civic tool for voter information in contemporary democracies. They typically present voters with a set of policy statements, ask them to express agreement or disagreement and then match their responses against the documented positions of competing parties or candidates. VAAs have been used by millions of citizens worldwide and have generated a substantial research literature on their effects on participation, vote choice and political knowledge ( Walgrave, Nuytemans, & Pepermans, 2009; Alvarez et al., 2014; Garzia & Marschall, 2014).

For our purposes, however, VAAs have two limitations that make them unsuited to primary elections. First, they focus on programmatic alignment, which is often the least differentiating dimension within a single party. When two candidates of the same party agree on most policy positions, a programmatic VAA cannot meaningfully distinguish them. Second, VAAs require advance work by their developers –typically academic teams, media or civil society organisations– to position each candidate on each policy item. This advance work is feasible for general elections, where the same parties compete repeatedly, but rarely available for primary elections, which are often called with limited notice and concern candidates whose positions have not been systematically documented.

Therefore, our tool is not a VAA. It does not match users to candidates. It produces structured analytical profiles that the user reads and interprets. The dimensions covered go beyond programmatic alignment to include leadership style, integrity, electoral potential, internal alliances, and (optionally) behavioral patterns. The advance work is performed at runtime by the language model, drawing on indexed public information.

### 2.3 AI-assisted deliberation: Opportunities and risks

The recent availability of large language models with web-search capabilities has opened a new space for civic tools ( Bender et al., 2021; Gemenis, 2024). These models can synthesise large amounts of public information on demand, adapt their output to the user’s level of expertise, sustain dialogue rather than delivering single answers, and operate in dozens of languages. For voter information specifically, the implications are significant: the bottleneck of advance curation that constrained VAAs is partially relaxed and the personalisation of informational flows becomes feasible at scale.

But the risks are equally significant ( Weidinger et al., 2022). Language models are known to produce confidently stated false information or “hallucinations”, to exhibit systematic ideological biases that vary by training and reinforcement, to generate persuasive content optimised for engagement rather than accuracy and to influence user beliefs in ways that are not always transparent ( Goldstein et al., 2023; Motoki, Pinho Neto, & Rodrigues, 2024; Salvi et al., 2024). When applied to electoral contexts –a field where the integrity of information has direct democratic consequences– these risks demand careful design.

The toolkit described in this paper is conceived as an attempt to harness the opportunities of conversational AI for primary-election information while mitigating the risks through explicit prompt-level constraints. Rather than fine-tuning a model or building a dedicated application, we use the more flexible and accessible approach of prompt engineering: a publicly readable set of instructions that any user can paste into the model of their choice, audit, modify and redistribute.

## 3. Implementation

### 3.1 Overall architecture

The toolkit consists of three text documents written in markdown, intended for direct copy-and-paste use. They are designed to be readable by humans (so users can audit and modify them) and parsable by language models (so they produce consistent behaviour across systems).

• **The main prompt** (prompt_candidate_analysis_v3–3.md) contains the analytical instructions. The user pastes this into a conversation with a language model and the model then asks structured questions and produces the analysis.
• **The external evaluator prompt** (external_evaluator_prompt_v1.md) contains instructions for auditing an output of the main prompt. The user pastes this into a separate conversation (ideally in a different language model), pastes the analysis to evaluate, and receives a structured audit report.
• **The methodological guide** (methodological_guide_v3–3.md) documents the design rationale, decisions, limitations, and recommended uses for distinct user types.

All three documents are released under MIT license and archived in Zenodo with persistent DOI.

### 3.2 Design principles

**Figure 1.**
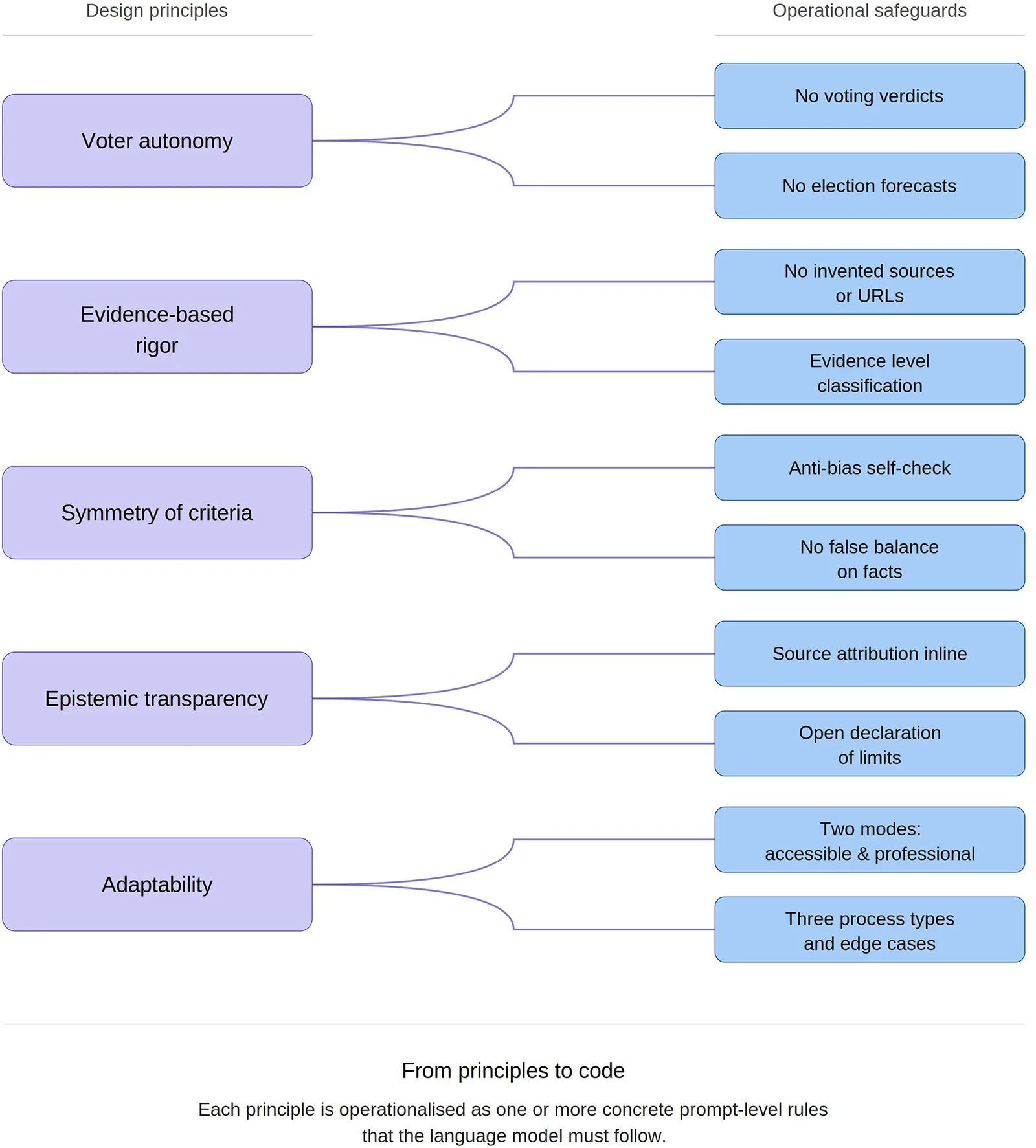
PRIMARYCAN’s five design principles and their operational safeguards. Each principle (left column) is encoded as one or more concrete prompt-level rules (right column) that the underlying language model must follow. The mapping makes principles auditable: any deviation in the output can be traced back to a specific safeguard, and any new safeguard can be motivated from the principles.

Five principles structure the design across all three documents: voter autonomy, evidence-based rigor, symmetry of criteria, epistemic transparency and adaptability without dilution. Each principle is encoded as one or more concrete prompt-level rules that the underlying language model must follow. The relationship is bidirectional: principles motivate safeguards, and safeguards in turn make principles auditable (see Figure 1). The principles are configured as it follows:

1. **Voter autonomy.** The toolkit informs voters; it does not recommend candidates. Voting verdicts and election forecasts are prohibited. When users press for a forecast or recommendation, the model redirects: voting is the user’s decision, prediction is something language models do poorly and the user’s own situated knowledge of the context is more reliable than the model’s. The tool’s role is to enrich criteria, not to substitute them.
2. **Evidence-based rigor.** All factual claims must be attributed to verifiable public sources. URLs are never invented. The model is instructed to declare evidence levels (abundant, moderate, scarce) for each candidate and adjust the analysis accordingly. Where indexed search is structurally limited (small municipalities, minor parties, less digitally mediatised contexts), the model declares this explicitly rather than producing complete-looking but speculative analyses.
3. **Symmetry of criteria.** All candidates are evaluated by the same standards, with the same kinds of evidence and the same analytical depth. Symmetry is of criteria, not necessarily of content: if one candidate has an open judicial case and another does not, both are analysed under the same criteria, but the result reflects the factual difference. This avoids the symmetric errors of bias (treating candidates differently based on non-evidentiary reasons) and false balance (presenting as comparable things that are not).
4. **Epistemic transparency.** The model distinguishes between facts, interpretations, and tentative hypotheses, marking the distinction either explicitly (professional mode) or through natural language cues (accessible mode). It declares its limits openly: when it cannot verify, when sources are dubious, when data are missing.
5. **Adaptability without dilution.** The toolkit operates in two modes: an accessible default for general voters, and a professional mode on explicit request for journalists, researchers, and party professionals. It detects the user’s language and adapts. It distinguishes three process types (active primary, retrospective analysis of a past primary, hypothetical simulation) with distinct rules for each. It handles edge cases (uncontested elections, runoffs, weighted-vote systems) with specific protocols. Throughout, the core principles above remain invariant.

### 3.3 Two analytical modes

The **accessible mode** is the default. It produces narrative profiles of approximately 120–150 words per candidate, organised around questions a real voter asks: who are they, what are they known for, what is their main strength, what is their main weakness. The output uses everyday vocabulary, short paragraphs, and natural source attribution. It includes a simple comparison table, a “what this comes down to” section that helps the voter structure the choice without recommending one, a deepening menu in natural language with candidate-specific suggestions and a brief disclaimer.

The **professional mode** is activated only on explicit user request (with phrases such as “I want a detailed analysis” or “I’m a researcher and need depth”). Self-identification as a journalist or researcher in the opening greeting is informative but does not by itself activate professional mode; the user must request the depth explicitly. Professional mode produces a structured analysis with six analytical blocks:

• **Block A** – Identity and trajectory: full name, age, current position, party, key milestones, territorial anchoring, education and prior experience.
• **Block B** – Discourse and communication: verified accounts and aggregated public data, dominant discursive axes with concrete recent examples, communicative registers and preferred formats, audience adaptation, dialectical performance, footprint in media agenda.
• **Block C** – Capabilities and performance: demonstrated management capacity, observable leadership style, teamwork, management of past crises and errors, resistance to prolonged wear.
• **Block D** – Consistency and integrity: ideological evolution, consistency between discourse and practice, ethical integrity, relationship with media and criticism, international projection.
• **Block E** – Political capital and electoral potential: internal alliance network, external support, reputation among different audiences, electoral potential adapted to the foreseeable horizon, compatibility with coalition.
• **Block F** – Advanced behavioral indicators (only on explicit request): democratic disposition, leadership style, observable behavioral patterns in six categories (public self-reference, instrumentalisation of relationships, response to criticism, public veracity, use of power, treatment of opponents).

Professional mode adds a seven-dimension comparative table, a balanced final reflection, a deepening menu with fixed dimensions and candidate-specific subsection, proactive second-order questions, an invitation to reflect on own criteria, and a formal limitations disclosure.

The choice of accessible mode as default emerged from controlled testing. Earlier versions of the toolkit, designed primarily for political science rigor, produced outputs that were rich but long and technical. Testing showed that accessible-mode outputs delivered the essential information in roughly half the length, with significantly higher readability and no loss of factual rigor. The design assumption is that an exhaustive analysis nobody finishes reading is worse for democratic decision-making than a shorter analysis people actually read.

### 3.4 Three process types

The toolkit distinguishes three types of process and adapts accordingly:

**Active primary**: a contest currently underway or upcoming. The analysis is forward-looking and decision-supporting. Speculation about non-active scenarios is avoided.

**Retrospective analysis**: a past primary the user wants to understand. The analysis reorients from “information for voting” to “explanation of the result”. This type is especially vulnerable to hindsight bias –the cognitive tendency to read past events as more inevitable than they were ( Fischhoff, 1975). The toolkit incorporates mandatory safeguards: recall the original uncertainty (full polling trajectory, not only final polls), distinguish structural from contingent factors, avoid inevitabilist framing, acknowledge alternative scenarios, beware polling selection bias, and close with an explicit anti-determinism line.

**Simulation**: a hypothetical exercise involving real candidates in scenarios that are not actually happening. The analysis is clearly marked as hypothetical throughout. Contingent projections of behaviour are permitted (“if they ran in this context, their trajectory suggests they would tend to …”). Outcome predictions are not.

Mixed cases (real candidates combined with added hypothetical ones) follow real-process rules for the real core and explicit marking for the hypothetical additions. The model judges case by case whether requested simulations are legitimate, using orienting criteria around apparent purpose, respect for sensitive situations, treatment of minors and deceased persons and analysis of non-political figures.

### 3.5 Edge cases

Three edge cases are explicitly handled in PRIMARYCAN’s v3.3:

**Single-candidate (uncontested) elections.** When only one candidate is running, the comparative architecture of the prompt does not apply. The model detects the situation, communicates it explicitly, reorients to individual analysis, and offers three balanced readings of the lack of competition: a positive reading (consensus around the candidate), a critical reading (barriers to opposition reduce democratic quality) and an intermediate reading (uncontested elections are common in some institutional contexts and reflect specific dynamics rather than necessarily indicating anomaly). It also discusses critical voting options (blank vote, abstention, negative vote where allowed) and acknowledges that the voter’s decision changes nature: from choosing among options to ratifying or not.

**Multi-stage primaries (runoffs).** When the analysed round is a runoff, the toolkit requires explicit vote-transfer analysis: which candidates were eliminated in the previous round, what percentage they received and how that vote may plausibly redistribute. The analysis identifies decisive levers (which voter segments, which campaign dynamics, which transfer patterns) without forecasting the outcome. It pays particular attention to substantive differentiation between finalists with similar profiles (same party faction, same demographic background), where surface features may obscure real differences.

**Special voting systems.** When the electoral system has notable peculiarities –weighted suffrage (different sectors weight differently in the result), delegate voting, mixed primaries with members and supporters weighted differently, endorsement requirements, ranked-choice voting– the toolkit explains the system to the user and indicates what it implies for reading the result. This addresses a finding from testing where systems with peculiar weighting structures significantly affect the strategic structure of the contest in ways that are easily missed.

### 3.6 The permitted-vs-forbidden distinction

Earlier versions of the toolkit had a blanket prohibition on “voting verdicts” that was sometimes interpreted by the model as also prohibiting useful analytical work that is not forecasting (such as analysing vote transfer dynamics in runoffs). Version 3.3 introduces an explicit distinction:

• **Forbidden**: predicting who will win, projecting vote percentages for upcoming elections, asserting outcomes of races that have not yet occurred.
• **Permitted and encouraged**: analysing the plausible dynamics that will likely shape the result. This includes identifying structural factors at play, analysing vote transfer dynamics in multi-round elections, identifying decisive variables (“levers”) and mapping uncertainties the user may be better positioned to resolve from their own knowledge.

The line is between *prescribing an outcome* (forbidden) and *describing the field of forces* (permitted). Such analysis ends by returning agency to the user.

### 3.7 The external evaluator

Testing revealed that internal self-evaluation by the model that produced the output has structural limits: some systematic errors (citation format, terminology choices, hindsight framing) escape the producer model’s own self-check, particularly when those errors are due to features of the producer model’s interface rather than its reasoning. To address this, v3.3 introduces a companion external evaluator prompt.

The evaluator is a separate prompt designed to be run in a different language model than the one that produced the output. It receives the analysis as input and produces a structured audit report covering critical findings (voting verdict avoidance, no invented content, criteria symmetry, process-type consistency, hindsight-bias safeguards, vote-transfer analysis in runoffs, balanced readings in single-candidate cases), important findings (mode consistency, citation format, evidence proportionality, loaded language, terminology sensitivity, felt fairness), recommended findings, specific concerns, and a synthesis with recommendation (disseminate / revise / rewrite) and confidence level.

The evaluator is most useful for journalists checking outputs before publication, researchers validating outputs before academic citation and educators training students in critical reading of AI-generated content. It is not a substitute for human expert review but a complement that catches a class of errors that producer self-evaluation misses.

### 3.8 Source attribution: a model-agnostic format

A key implementation detail emerged from testing. Different language models have different internal citation systems: some convert search results into clickable inline references, others produce parenthetical citations in prose, others produce bibliography-style lists at the end. A prompt that assumes one style produces inconsistent output across models.

Version 3.3 specifies a model-agnostic format. For every relevant factual claim, the model includes a parenthetical attribution with source and date in the body of the text –e.g., “Cassidy was one of seven Republican senators who voted to convict Trump in 2021 (Wikipedia, April 2026)”. Markdown links to URLs are allowed only when the model can recover verifiable URLs from its web search. The model is explicitly forbidden from inventing URLs: if it cannot recover a real one, it uses parenthetical attribution without a link.

This implementation detail matters because it directly affects the verifiability of outputs. A user who reads “according to Reuters” can search for the relevant Reuters article. A user who reads a hallucinated URL is led to believe the source is more accessible than it actually is.

### 3.9 Behavioural patterns, not clinical labels

An early design decision worth highlighting concerned the analysis of personality-related dimensions. The political psychology literature includes substantive frameworks for personality assessment in political leaders, including the Dark Triad/Tetrad (narcissism, Machiavellianism, psychopathy, sadism; see Paulhus & Williams, 2002; Nai & Toros, 2020). However, rigorous application of these frameworks requires validated psychometric instruments administered directly to the person, and their casual application to public figures based on observed behaviour conflates clinical assessment with informed public commentary.

The toolkit explicitly rejects this framework and replaces it with an analysis of *observable behavioural patterns* in six categories: public self-reference, instrumentalisation of relationships, response to criticism, public veracity, use of power and treatment of opponents. For each pattern, the model is required to provide at least one verifiable example with source, to note counter-indicators where they exist and to use descriptive formulations (“shows a pattern of …”) rather than identitarian ones (“is a narcissist”). This shift from clinical labels to behavioural patterns is both ethically more defensible and empirically more robust.

The model is also instructed to recognise that the absence of counter-indicators may reflect either non-existence or absence of documenting material –an asymmetry the analysis should not over-read.

### 3.10 Iterative design and version history

The toolkit’s current state is the result of six successive versions developed over a compressed timeframe of intensive iteration:

• **v1.0**: initial three-level structure (descriptive, evaluative, advanced).
• **v2.0**: introduction of six analytical blocks; synthetic-profile-then-deepen format; anti-bias self-check protocol.
• **v3.0**: rewriting of the prompt in English (with multilingual user interaction preserved); introduction of the real-process-vs-simulation distinction; consolidation of the six blocks; introduction of four-layer temporal weighting; replacement of Dark Triad nomenclature by the behavioural-patterns framing; optimisation for Claude, ChatGPT, and Gemini.
• **v3.1**: inversion of the default to accessible mode (with professional mode on explicit request); two parallel output architectures; Block F made available in both modes; model-agnostic source citation format; explicit grouping of candidate-specific deepening suggestions; recognition of counter-indicator asymmetry.
• **v3.2**: addition of the third process type (retrospective analysis) with mandatory hindsight-bias safeguards; addition of the terminological sensitivity instruction; expansion of the final disclosure in professional mode.
• **v3.3** (current): explicit handling of multi-round primaries, single-candidate elections, special voting systems, and limits of indexed search; refinement of the permitted-vs-forbidden distinction; prioritisation of the self-check in three tiers; addition of the external evaluator prompt as companion document.

Each version was tested against representative cases before progressing. The full version history is documented in the changelog of the methodological guide. The toolkit performed robustly across language (Spanish, English), mode (accessible, professional), and process type (active, retrospective, simulation). It also performed reasonably in cases outside its explicit design scope (non-partisan elections, single-candidate situations, runoffs, weighted-suffrage systems), thanks to the generality of its underlying principles. Many of the v3.3 enhancements derive from formalising what the toolkit was doing well by spontaneous extension, rather than from correcting failures.

The most consistent improvement pathway across iterations was specification: where the prompt was abstract or general, the model produced inconsistent output across executions; where the prompt provided concrete rules, examples, or formats, the model produced consistent output. This finding aligns with the broader prompt-engineering literature on the importance of specificity ( Liu et al., 2023; White et al., 2023).

The most consistent failure mode was the leakage of producer-specific features into the output (such as the citation system in Use Case 2). This class of error tends to escape model self-evaluation because the producing model considers them normal –they look like the citation system the producer always uses. The external evaluator addresses this class of error by being run in a different model that does not share the producer’s interface assumptions.

## 4. Discussion

The toolkit makes three contributions. First, *substantively*, it offers a freely usable, adaptable, multilingual tool for voter information in primary elections –a context where the existing civic infrastructure (VAAs, fact-checkers, professional media) is structurally weaker than in general elections. Second, *methodologically*, it demonstrates one approach to scaffolding conversational AI for democratic deliberation: explicit ethical and epistemic constraints, layered defaults that respect user expertise, edge-case handling that emerges from underlying principles and an external evaluator that addresses the structural limits of self-evaluation. Third, *conceptually*, it operationalises a distinction between forbidden prediction and permitted dynamics analysis that may be useful beyond its specific application: the line between describing the field of forces and prescribing an outcome.

On the other hand, several limitations should be acknowledged. Although the toolkit mitigates bias and hallucinations by requiring verifiable sources and forbidding URL invention, the risk is reduced but not eliminated. Users producing high-stakes content –publication, campaign materials, formal advice ( Makse & Zava, 2024) – should treat the output as raw material for human verification, not as final analysis. Human expert review remains the strongest validation, particularly for high-stakes use. Also, as a concept, the toolkit has been tested by its designers in controlled cases. It has not yet been validated through use by real voters in real primary elections, nor through a systematic between-model comparative study with external rating of outputs. These are the most important pending steps.

Beyond this, the development of this toolkit raises a question that exceeds its immediate technical scope: what is the appropriate role of AI in democratic deliberation? Two extreme positions are unattractive. On one hand, AI tools that decide for voters –recommending whom to vote for, predicting outcomes, ranking candidates by AI-derived scores– substitute machine output for civic agency and risk producing a passive, dependency-prone electorate. On the other hand, refusing AI any role in democratic deliberation forfeits real opportunities to address well-documented information asymmetries that affect electoral participation and decision quality ( Buhmann & Fieseler, 2023; Jungherr & Rauchfleisch, 2025).

The toolkit takes a middle position: AI as *deliberation infrastructure*, not as decision-maker. It systematises information, structures analysis, raises questions the voter might not have considered, declares its limits, and returns agency. The voting decision remains the voter’s. This position rests on the empirical claim that better information improves decision quality ( Lupia, 1994) and on the normative claim that voter autonomy is constitutive of democratic legitimacy ( Christiano, 1996; Estlund, 2008).

Whether this middle position is sustainable in practice depends on factors beyond the toolkit’s design: how users actually engage with it ( Gulati et al., 2024), whether competing tools displace it with more directive alternatives, whether AI providers maintain web-search and reasoning capabilities at accessible cost. These factors are not under the toolkit’s control. What the toolkit can do –and attempts to do– is demonstrate that a more democratically respectful design is feasible. In this sense, developing, testing and sharing open-source prompts is a way to foster citizen science ( Stilgoe, 2009; Amoore et al., 2025).

## 5. Conclusions

We have described an open-source conversational AI toolkit for citizen deliberation in partisan primary elections. The toolkit consists of three components: a main analytical prompt with two modes and three process types, an external evaluator prompt for auditing outputs, and a methodological guide. It implements explicit safeguards against common pitfalls in algorithmic political analysis, handles a range of edge cases, and is freely usable by any voter with access to a major conversational AI system. The toolkit’s central design wager is that AI can serve democratic deliberation as infrastructure rather than as decision-maker –systematising information, structuring analysis, declaring limits, and returning agency to the voter. Whether this wager is borne out in practice depends on factors beyond the toolkit’s design: empirical validation with real users, sustained capabilities of underlying AI systems and the broader information ecosystem within which it operates.

We release the toolkit as an open resource and invite adaptation, criticism and use across different contexts. The next steps are empirical: real-world testing in real primaries, comparative studies across models and countries, and the development of a sibling toolkit for non-partisan electoral processes. We expect the toolkit’s most useful contributions to emerge not from the design alone, but from the encounter between the design and the actual practices of voters, journalists, researchers and educators who may choose to use it.

## Ethics and consent

Ethical approval and consent were not required.

## Declaration of AI use

The development of this toolkit benefited from the iterative collaboration between the authors and conversational AI assistants used as design interlocutors during the prototyping phase. We explicitly disclose that the toolkit’s prompts were drafted, tested, and refined through extended iterative dialogue with Anthropic’s Claude (Opus 4.7 version). The substantive design decisions, the conceptual architecture, the ethical safeguards, the choice of cases and the final form of the toolkit are the authors’ responsibility. The AI assistant served as a sparring partner for prompt drafting, edge-case identification, rapid prototyping and also for proofreading this manuscript.

## Data Availability

- Source code available from: https://github.com/rvillaplana/PRIMARYCAN-Prompt

- Archived software available from: The complete toolkit (main prompt v3.3, external evaluator v1.0, methodological guide v3.3) and extended data are archived at Zenodo under the DOI https://doi.org/10.5281/zenodo.20053952. - License: MIT. The toolkit requires no installation. It is used by copying the relevant prompt text into a conversation with a large language model that has active web-search capability (currently Claude, ChatGPT, Gemini and similar systems).
