# Supplementary material for: A conversational AI toolkit for citizen deliberation in party primary elections
Source: Open Res Eur. 2026 Jun 13;6:201. [Version 1] doi: 10.12688/openreseurope.24102.1 (PMC13562963; doi:10.12688/openreseurope.24102.1)
Supplement: Peer review history

# Reviewer report: Reviewer response for version 1

**DOI:** 10.21956/openreseurope.26091.r76062

**Published:** 2026-09-07

**Authors:** Enrico Biale

**Affiliations:** 1 University of Piemonte Orientale, Vercelli, Italy

This article describes the development of an AI tool that aims to provide citizens with information about the candidates in a party’s primaries. The authors rightly recognize that primaries have increasing weight within the selection of political personnel but do not receive the same attention from the media, with the consequence that citizens are poorly informed about the candidates and are unable to exercise full control over their choice. This, on the one hand, risks confirming the limits of similar procedures that give greater weight to people who are members of and active within a party and, on the other, is in tension with the attempt by parties to extend primaries also to non-members, since it limits their role. Since Voting Advice Applications cannot be used for primaries, the authors develop a prompt that is compatible with the main chatbots (Claude, Gemini, ChatGPT) and provides an analysis of the candidates running in a primary election. The interesting aspect of the tool is that it makes it possible to provide an analysis of the candidate and a verification, which the authors rightly suggest carrying out with an alternative chatbot, of the chatbot’s analysis of the candidate, so as to increase citizens’ control and the model’s accuracy.

 This proposal is certainly very innovative and interesting. It fills a gap within democratic systems and fits into that strand of research which argues that AI can facilitate the deliberative process, by providing a sort of infrastructure, but cannot replace or corrode it. There are, however, some aspects that could be further clarified in order to make this proposal even more convincing:

 1) the authors seem to suggest that the tool will provide citizens with a written assessment of the candidates. It is not clear whether it is possible, as often happens with a chatbot, to interact with the tool in such a way as to ensure a more active role for citizens and facilitate the deliberative process at which the model seems to aim. If interaction is envisaged, this should be explicitly clarified in the text. If it is not envisaged, it would be useful to explain why this option was not included, since it seems the most natural way to facilitate a deliberative process.

 2) Such a tool seems to require a certain degree of technical competence from users, and this could increase informational asymmetries between expert and non-expert users. If this is not the case, the authors should clearly explain that use of the tool does not require any technical competence or, in any case, requires only very limited competence. If there are asymmetries in use among users who have different levels of experience, it would be useful for the authors to clarify how this does not negatively affect the role of the tool and the levelling function it should have.

 3) The authors rightly argue that the tool must inform but not manipulate/influence citizens’ choices, so that they retain full control over the decision-making process. For this reason, a predictive function was explicitly excluded. Although I understand the reason for this choice, it excludes a very important dimension within a primary election, namely the chances of success of a certain candidate in the general election. In many cases, citizens legitimately engage in forms of strategic calculation that lead them to choose a less preferred candidate who, however, has a greater chance of success. Excluding this possibility seems to limit the scope of the tool without ultimately excluding these forms of strategic calculation. Citizens will, in fact, continue to engage in them but will not be accurately informed, thus risking making suboptimal choices. It would be interesting to understand whether developments of the tool could fill this gap and provide reliable information also with respect to this dimension.

 4) The idea of AI as a deliberative infrastructure is very interesting but currently undertheorized and only briefly mentioned in the concluding part of the paper. It would be interesting to develop this part, perhaps moving it to the introductory section and clarifying how this tool contributes to the idea of AI as a deliberative infrastructure.

Is the rationale for developing the new software tool clearly explained?

Yes

Is the description of the software tool technically sound?

Yes

Are sufficient details of the code, methods and analysis (if applicable) provided to allow replication of the software development and its use by others?

Yes

Are the conclusions about the tool and its performance adequately supported by the findings presented in the article?

Yes

Is sufficient information provided to allow interpretation of the expected output datasets and any results generated using the tool?

Yes

Reviewer Expertise:

Democratic Theory, Political Theory

I confirm that I have read this submission and believe that I have an appropriate level of expertise to confirm that it is of an acceptable scientific standard.

# Reviewer report: Reviewer response for version 1

**DOI:** 10.21956/openreseurope.26091.r76956

**Published:** 2026-07-22

**Authors:** Ignacio Jesús Serrano Contreras

**Affiliations:** 1 Universidad de Granada, Granada, Spain

The ideas reflected in this project are highly interesting, especially if we accept that these are debates, we must primarily address within academia-either to study what they entail or to provide technical contributions to these dilemmas. Nevertheless, there are a few points that warrant reflection: - Since this case involves a Software Tool Article, it is understandable to focus on the tool's design. However, it would be highly valuable to include data (both quantitative and qualitative) regarding the five cases that interacted with the developed toolkit. Therefore, a brief or explanatory section could be included in the appendices to detail, for example, which models were used, as well as the inconsistencies or failures encountered.

 - I consider that the key issue lies within the algorithms themselves-specifically the LLMs employed-and especially the prevalence of the flaws already inherent to commercial models. A prompt, no matter how symmetric it is, risks operating on training foundations that already discriminate from the outset, particularly against outsiders, populists, or minority factions. I assume that within this methodological discussion, this baseline bias could be bypassed through testing across local, commercial, or open-source models, as well as considering (which I assume is the ultimate goal) the implementation of part of these redefinition tasks by fine-tuning a specific model. All of this, even while always considering the inherent bias, would constitute the most feasible method to deal with potential biases, especially regarding issues like indexing or real-time web search prioritized by the models used. - Following this, it is necessary to consider how these conversational chats will deal with leading or biased questions, the use of reverse rhetoric, or other scenarios where, depending on the model, the reliability of the response would also be affected. This aspect is complex to avoid-even more so than issues like indexing-but it also enters a highly interesting ethical ground. Thus, it would be valuable to include whether safeguards for conversational development have been foreseen in this regard.

 - On another note, the enrichment that .md brings over .txt is interesting, but the preponderance of semantic hierarchies provided by elements such as # or **, etc., must be considered. Therefore, I also recommend a technical justification to enrich this point.

 - Lastly, regarding accessibility and following up on the previous point, for the average user who is unfamiliar with the .md format, it can represent a barrier. If an interface or a redirection to a specific model that copies and pastes the prompt is already planned, it would be interesting to include it. In conclusion, it is clear that we are dealing with a highly timely topic that, as stated, must be debated and reflected upon, in addition to-as in this specific case-providing proposals from academia. Thus, this work has great potential, and by addressing a few details, the current deepening of this study will yield debates that are more than pertinent.

Is the rationale for developing the new software tool clearly explained?

Yes

Is the description of the software tool technically sound?

Yes

Are sufficient details of the code, methods and analysis (if applicable) provided to allow replication of the software development and its use by others?

Partly

Are the conclusions about the tool and its performance adequately supported by the findings presented in the article?

Yes

Is sufficient information provided to allow interpretation of the expected output datasets and any results generated using the tool?

Partly

Reviewer Expertise:

Political communication; Algorithms; Public Opinion

I confirm that I have read this submission and believe that I have an appropriate level of expertise to confirm that it is of an acceptable scientific standard.
